# Assessment of Oral Microbiome Changes in Healthy and COVID-19-Affected Pregnant Women: A Narrative Review

**DOI:** 10.3390/microorganisms9112385

**Published:** 2021-11-19

**Authors:** Andrea Butera, Carolina Maiorani, Annalaura Morandini, Manuela Simonini, Arianna Colnaghi, Stefania Morittu, Stefania Barbieri, Maria Ricci, Gaetano Guerrisi, Daniela Piloni, Roberta Cimarossa, Barbara Fusaro, Antonia Sinesi, Ambra Bruni, Andrea Scribante

**Affiliations:** 1 Unit of Dental Hygiene, Section of Dentistry, Department of Clinical, Surgical, Diagnostic and Pediatric Sciences, University of Pavia, 27100 Pavia, Italy; 2 Member Association: “Mamme & Igieniste”, 24125 Bergamo, Italy; dr.annalauramorandini@gmail.com (A.M.); manuelasimonini@libero.it (M.S.); colnaghi.arianna@gmail.com (A.C.); morittustefania3@gmail.com (S.M.); stefaniabarbieri@me.com (S.B.); maridental70@gmail.com (M.R.); gaetanoguerrisi@libero.it (G.G.); piloni.daniela@libero.it (D.P.); roberta.id@cimarossa.it (R.C.); bfusaro@libero.it (B.F.); antonia.sinesi@gmail.com (A.S.); ambrabruni@libero.it (A.B.); 3 Unit of Orthodontics and Pediatric Dentistry, Section of Dentistry, Department of Clinical, Surgical, Diagnostic and Pediatric Sciences, University of Pavia, 27100 Pavia, Italy; andrea.scribante@unipv.it

**Keywords:** oral microbiome, pregnancy, periodontitis, oral health, dentistry, dysbiosis

## Abstract

During pregnancy, there are several metabolic changes and an alteration in the composition of microorganisms that inhabit the oral cavity, with an increase in pathogenic bacteria that promote the onset of gingival diseases. This review is based on research in reference to the PICO model (Problem/Intervention/Comparison/Outcome), related to changes in the oral microbiome of pregnant women and possible oral consequences in patients with COVID-19. The results showed a growth of some pathogenic bacteria in pregnant women, including *Aggregatibacter actinomycetemcomitans* and *Fusobacterium nucleatum*, and the selective growth of the *Prevotella intermedia*, *Porphyromonas gingivalis* and *Tannerella* species, probably due to the fact that these bacteria use progesterone as a source of nutrition. These same bacteria are implicated in the development of periodontal disease. Periodontal pockets have bidirectional interactions between the oral cavity and the systemic circulatory system through the peripheral gingival blood vessels. The affinity of the SARS-CoV-2 virus to specific membrane receptors is now clear, and could involve the internal and external epithelial lining or the fibroblasts of the periodontal ligament. According to the results of the present review, the control of oral microbiome changes during pregnancy would be welcomed. The use of probiotics could help clinicians manage pregnant patients, reducing inflammatory indexes. Future studies should focus not only on changes in the level of the oral microbiome in pregnancy or the correlation between periodontal disease and COVID-19, but also on oral changes induced by both clinical situations.

## 1. Introduction

The oral microbiota is a set of microorganisms that lives in symbiosis in an organism, similar to a photographic shot capturing a population of microscopic organisms residing in a space delimited to a shot chosen arbitrarily. This concept has also been suggested by several studies, including recent studies [1,2].

These micro-organisms help to prevent the development of diseases, protect the oral cavity and maintain the homeostasis of the organism. The genes that the organism can express define the oral microbiome. In healthy patients, the oral microbiome includes up to 600 species, such as *Steptococci*, *Lactobacilli*, *Staphylococci* and *Cotynebacteria.* However, the condition of eubiosis changes during pregnancy, with an increase of pathogenic taxa, including high levels of Poprhyromonas gingivalis and Aggregatibacter actinomycetemcomitans. These bacteria can promote the onset of gingival disease and the development of periodontal disease [3].

Many of the metabolic changes characteristic of pregnancy are similar to those of metabolic syndrome, presenting weight gain, high fasting blood sugar levels, insulin resistance, glucose intolerance, low-grade inflammation and changes in metabolic hormone levels. Such endocrine-metabolic-immune alterations correlate with significant microbial changes at different sites of the organism [4,5]. During pregnancy, in fact, there are numerous changes, such as the swelling of endothelial cells, the increase of platelets and the adhesion of leukocytes to the walls of blood vessels, the formation of microthrombi and the increase of vascular permeability, due to hormones estrogen, progesterone chorionic, and gonadotropin. Estrogen and progesterone are associated with inflammatory phenomena, variations in vascular responses and connective tissue turnover in periodontal tissue [6,7]. The high levels of estrogen modify the oral mucosa: The gums have a hypersensitivity to local factors, such as bacterial plaque, calculus, prosthetics and overdue reconstructions. The response occurs with an increase in volume, bleeding and edema until the appearance, in some cases, of periodontal disease, which tends to regress after the end of pregnancy. These are well-documented changes that occur at the level of the oral microbiome, with an increase of anaerobic and aerobic bacteria, such as *Bacteroides melaninogenicus*, *Prevotella intermedia* and *Porphyromonas gingivalis* [4,8,9,10].

In addition, changes in the oral microbiome appear to be associated with glycemic control: More *Proteobacteria* and less *Firmicutes* and *Leptotrichia* have been found in women with gestational diabetes [11]. Over the years, the latter has become one of the most common complications during the course of pregnancy, increasing the risk of gestational hypertension, fetal dysplasia, restriction of fetal growth, miscarriage and premature childbirth, polyhydramnios, macrosomia, advanced diabetes and obesity [12].

In light of the changes in the oral microbiome in pregnant women, which predispose and encourage the development of gingival problems, it is useful to investigate any correlations with COVID-19. In fact, in patients affected with COVID-19, there is an increase in the *Prevotella*, *Fusobacteria* and *Veilonella* species, involved in the development of periodontal disease. This finding could represent a link between periodontitis and COVID-19 [13]. In most cases, patients with COVID-19 and whose course increases in complexity are those with different systemic diseases, such as cardiovascular problems and diabetes, often associated with an increase in some pathogenic bacteria, such as *Fusobacterium nucleatum*, *Prevotella intermedia* and *Porphyromonas gingivalis*, that favor the progression of periodontal disease [14]. 

## 2. Material and Methods

### 2.1. Focused Questions

How does the oral microbiome change in pregnant women? What are the possible oral findings in pregnant women with COVID-19?

### 2.2. Elegibility Criteria

First, we analyzed the studies in accordance with the following inclusion criteria:

*Type of studies*. Case-control, cross-sectional, cohort studies, clinical trials and reviews.

*Type of participants.* Pregnant women and pregnant women with COVID-19.

*Type of interventions*. Case-control, cross-sectional, cohort studies and clinical trials that have evaluated the major changes in the oral microbiome level in pregnant women and possible oral correlations with COVID-19.

*Outcome type*. Changes in the oral microbiome in pregnant women and possible correlations with COVID-19.

In the second phase, we included only those studies that met all the inclusion criteria, that is to say, the analysis of the selected studies according to the exclusion criteria: (I) studies where the authors had not reported at least one of the parameters chosen as outcomes, (II) studies performed on participants with concomitant systemic pathologies/treatments that could have affected outcomes, (III) in vitro or animal clinical studies, (IV) studies conducted without the approval of the Ethics Committee. 

### 2.3. Search Strategy 

This narrative review is based on research in reference to the PICO model (Population, Intervention, Comparison, Outcome), identified through bibliographic research in electronic databases and by examining the bibliography of articles on Pubmed (MEDLINE) and Google Scholar. Initially, all study abstracts were taken into consideration, which evaluated the changes in pregnant patients’ oral microbiome and possible oral findings in pregnant women with COVID-19.

### 2.4. Research

We performed the search using the following keywords: “COVID-19” AND “pregnant women”, “COVID-19” AND “pregnancy”, “COVID-19” IN “pregnancy” AND “oral complications”, “oral microbiome” AND “pregnancy”, “oral microbiome” AND “COVID-19”, “pregnancy” AND “oral lesions”, “COVID-19” AND “oral cavity”, “COVID-19” AND “periodontitis”.

## 3. Synthesis of Results

In pregnant women, the presence of certain pathogenic bacteria such as *Treponema denticola*, *Fretibacterium* spp., *Prevotella_intermedia*, *Tanerella forsythia*, *Aggregatibacter actinomycentemcomitans* and *Porphyromonas gingivalis*, which are more prevalent in the early and middle stages of pregnancy, was reported. In addition, the presence of *Fusobacterium nucleatum* was reported, which may lead to complications during pregnancy, including miscarriage, intrauterine death, neonatal death, preterm delivery and premature rupture of the membranes. Other adverse events are low birth weight and pre-eclampsia [8,15,16].

Pregnant women are more prone to gingival inflammation, gingivitis and periodontal disease. Among these patients, other injuries were reported, such as morsicatio buccarum, oral candidiasis, pyogenic granuloma and benign migratory glossitis (Table 1).

### Risk of Bias 

The risk of bias could not be assessed due to insufficient detail. Table 2 shows the risk of bias of the main articles examined. This review presents a relatively moderate risk of bias.

## 4. Discussion

The oral cavity houses the second largest microbe, after the intestinal one, being the reservoir of over 600 bacteria species [10]. The composition of this microbiome is influenced by several clinical factors such as diabetes mellitus, atherosclerosis and cardio-vascular problems, autoimmune diseases, menopause and pregnancy [3].

The numerous physiological alterations that accompany pregnancy can significantly affect the state of oral health in the woman. Pregnancy is characterized by a series of morpho-functional modifications, determined by the interaction between the development of the product of conception in the growth phase and the progressive adaptation of the organism. In addition, the pregnancy period is accompanied by endocrine and oral changes due to the increase in hormone levels in plasma, which have negative effects on the periodontal health of the mother. Hormonal changes, both vascular and immunological, can generate in gingival tissues an exaggerated inflammatory response toward pathogenic microorganisms belonging to the oral biofilm [4,17,18].

According to estimates in the literature, 60–75% of pregnant women have gingivitis and, if present even before pregnancy, 50% of them develop periodontal disease. In general, it is estimated that 25% of pregnant women suffer from periodontitis [19]. Although there is no clear and well-validated association between periodontal disease and pregnancy, some factors justify its development. Focusing, however, on the changes that the state of pregnancy entails to the oral microbiome, it is easy to think that the breeding ground created for pathogenic bacteria can promote the onset of a tissue pathology. The oral microbiome that populates periodontal pockets is anaerobic, similar to the one that occurs when pregnant [20]. In addition, monitoring the periodontal condition of these patients is even more crucial, as several studies have revealed potential related risks, such as premature birth (at approximately the 37th week), low birth weight (2500 g) and preeclampsia/eclampsia (hypertension induced during pregnancy, high levels of protein in urine) [21].

During the second and third trimester of pregnancy, the onset of periodontal lesions is frequent. From an ethiopathogenetic point of view, the correlation between periodontitis and negative events associated with pregnancy is supported by some hypotheses. The first is based on the possibility that women with periodontitis are prone to frequent bacterial infections. The bacteria activate a cascade of inflammatory processes at the level of the placenta and the fetus, with the risk of pretermination delivery/the birth of underweight children. The second hypothesis is that periodontitis can cause a generalized increase in cytokines, substances with proinflammatory activity that cause alterations in the placenta and fetus. Among these, the reduced increase in the body weight of the unborn child and the development of premature uterine contractions, with the risk of preterm delivery/the birth of underweight children, are relevant [22,23].

Therefore, careful periodical controls with dental hygiene clinicians during pregnancy are strongly recommended to evaluate the inflammation of soft tissues and to plan non-surgical periodontal therapy or a domiciliary support with the correct use of electric tooth brushes, probiotics and natural compounds [1,9]. 

Thus, during pregnancy, there are numerous changes concerning the decrease in the number of neutrophils, the decrease of chemotaxis and phagocytosis, and a depressed antibody response and cell immunity, which causes the formation of an extremely varied subgingival biofilm. Several studies have been conducted to assess the composition of the oral microbiome during pregnancy and to highlight any differences [4,8,9,10]. 

A study of nine pregnant women in the first trimester in the Netherlands showed an increase in *Prevotella intermedia*, *Porphyromonas gingivalis*, *Treponema denticulae* and *Aggregatibacter actinomyctemcomitans* [13]. The selective growth of *Prevotella intermedia*, *Porphyromonas gingivalis* and *Tannerella* species was probably due to the fact that these bacteria use progesterone as a source of nutrition [24], but also because there are changes in the immune system and local changes at the gingival level, such as bleeding, which provide additional nourishment. In the case of periodontal disease related to pregnancy, anaerobic bacteria find favorable environments within periodontal pockets [25]. These results are in agreement with other studies that have highlighted the increase of *Porphyromonas gingivalis* and *Aggregatibacter actinomyctemcomitans* at the beginning and middle of pregnancy, along with an increase of *Streptococci*, *Staphylococci* and *Candida* [26].

This alteration in the oral microbiome, with an increase of pathogenic taxa, causes the activation of cell-mediated immunity and the production of interleukins, such as tumor necrosis factor and postaglandin-PGF2 [24]. These inflammatory mediators can promote adverse factors to childbirth, such as a premature birth or a low birth weight [27,28]. A case of a pregnant woman with gingivitis caused by *Fusobacterium nucleatum*, isolated at the level of the placenta and the newborn, ended with the birth of the dead baby [29].

Other oral health problems that may occur in pregnant women are caries and pregnancy oral tumors, such as the granuloma that usually appears in the second and third months of pregnancy. Progesterone works as an immunospressor on the gingival tissues of pregnant women, which leads to an increase in chronic tissue reaction with an exaggerated appearance of inflammation. 

Another point inherent in the possible oral problems and changes in the level of the microbiome residing in the oral cavity could be the development of the disease from COVID-19, whose severity seems related to the state of gingival health. Therefore, one of the objectives of this review was to assess the presence of a further oral change in pregnant women with COVID-19. Regarding this disease, there are not many findings in the literature, but it is known that COVID-19 leads to systemic changes and predisposes patients to opportunistic infections. 

In pregnant women, there were no cases of vertical transmission to the unborn child. In the scarce studies conducted to date, the results have demonstrated the favorable health of newborns and good recovery. Most studies have not reported major complications from COVID-19. Nevertheless, in some studies, hospitalizations in the ICU have been reported with the request of mechanical ventilation or O2 (7% of pregnant women needed to be admitted to intensive care and 9% needed O2) [30,31,32,33], or severe to critical conditions (2% to 9%) [34,35,36,37]. In addition, from the examined studies, a high incidence of C-sections and premature births, spontaneous miscarriages and the restriction of uterine growth has emerged [38,39,40,41]. 

Studies on the possible oral complications of COVID-19 in pregnant women have shown cases of dysgeusia or xerostomy, petechiae, reddish maculae and gums desquamate. Few data are available, however, on enamel defects, such as hypodontia, gyroversion, microdontia, enamel hypoplasia, enamel hypomineralization, impacked teeth, dens in den, dens invaginatus/evaginatus, taudorism and ghost teeth [42,43]. 

The quiescence time between the onset of systemic symptoms and oral lesions could be between 4 days before and over 12 weeks after the onset of systemic symptoms. One study reported that in three cases, oral lesions preceded systemic symptoms, and in four cases, oral and systemic symptoms appeared at the same time. These symptoms diminished between 3 and 28 days after the onset, with the help of different types of antidotes, including chlorhexidine mouthwash, nistatin, oral fluconazole, topical or systemic corticosteroids, systemic aciclovir, artificial saliva and photobiomodulation remedy (PBMT), depending on the etiology [44]. 

In addition, regarding gingival problems and changes in the composition of the subgingival plaque, periodontal pathogens, which are embedded into the lower respiratory tract, can lead to increased expression of ACE2 on the epithelial cells of the lower respiratory tract and thus promote the infectivity of SARS-CoV-2, as well as the epithelial mucous membranes of the oral mucosa [45]. Oral clinical manifestations from COVID-19 include ulcers, vesicles, bleeding and oral candidiasis involving the mucous membranes of the tongue, palate, lips, gums and cheeks [44]. However, it is unclear whether these symptoms are caused by the disease or an overlap with other microorganisms.

Considering changes in the level of the oral microbiome in pregnant women and the possible complications of COVID-19, it is necessary to frame the problem according to the emerging findings. During pregnancy, there is an increase in oral bacteria such as *Porphyromonas gingivalis, Treponema denticola, Prevotella intermedia, Tanerella forsythia, Aggregatibacter actinomycetemcomitans* and *Campylobacter rectus*, involved in the development and progression of gingival diseases [15]. Among these, the major findings during pregnancy are gingival hyperplasia (17.1%), morsicatio buccarum (10%), oral candidiasis (4.4%), pyogenic granuloma (3%) and benign migratory glossitis (2.8%) [5]. 

Although microbial diversity remains stable during pregnancy, there is an increase in bacteria belonging to the species of *Neisseria* spp., *Tremonema* spp. and *Pophyromonas* spp. that can induce pathological states [10]. Periodontal parameters such as plaque index, gingival index, probing pocket depth and gingival bleeding have been shown to increase during pregnancy. Pregnant women have a higher gingival index and higher indices such as probing pocket depth than non-pregnant women [16]. In addition, *Prevotella intermedia*, *Porphyromonas gingivalis* and *Fusobacterium nucleatum* are abundant during pregnancy, as reported by several studies, which have shown an association with the development of gingivitis. Subsequently, postpartum, there is a significant decrease of these same pathogenic species: *Aggregatibacter actinomycetemcomitans*, *Porphyromonas gingivalis* and *Tanerella forsythia* are greatly diminished after childbirth [8,46,47].

In particular, attention should be paid to the changes induced by *Fusobacterium nucleatum*. One study found the presence of *Fusobacterium nucleatum* in patients with COVID-19, suggesting the development of a bacterial bacteria due to bacterial translocation. The same pathogenic bacterium was found in the mucus of the colon, as well as in the bronchoalveolar washing fluid of patients with COVID-19 [48,49,50,51]. In addition, *Prevotella intermedia*, considered one of the main bacterial species implicated in acute periodontal lesions, was found in affected or suspected COVID-19 subjects. This could predispose individuals to necrotizing periodontal disease through bacterial co-infection propagated by the micro-organism itself [51]. One study, in fact, reported the case of a female patient with severe halitosis, erythematous and edematous gums and necrotic interdental papillae, resolved with antibiotic therapy and chlorhexidine and accompanied by the disappearance of the symptoms of suspected COVID-19. These findings contribute to our understanding of the role of bacterial coinfections in the severity of this respiratory syndrome [52]. Table 3 shows variations between the oral microbiome of a healthy patient and the oral microbiome of a patient affected by COVID-19 disease and a pregnant woman. 

Periodontal pockets have, in fact, bidirectional interactions between the oral cavity and the systemic circulatory system through the peripheral gingival blood vessels: in the case of SARS-CoV-2, the affinity of the virus to specific membrane receptors is now clear and this could involve the internal and external epithelial lining or the fibroblasts of the periodontal ligament [53,54].

Therefore, with the increase of the pathogenic bacterial load in pregnancy, which favors the onset of gingival diseases, attention must be paid to the restoration of favorable microbiological conditions. The use of probiotics for the restoration of a correct oral microbiome has been studied, with a reduction of the percentage of pathogens belonging to the orange complex, and a reduction of copies/microliters of *Prevotella intermedia* and *Fusobacterium nucleatum* [54]. In addition, the use of probiotics is useful in reducing certain clinical parameters, such as BoP, FMBS and mSBI [55]. This can also help the course of respiratory dysbiosis, which could be caused by poor oral hygiene, coughing, mechanical ventilation or conditions that put the oral microbiome in contact with the respiratory tract. To promote proper home hygiene, it is also advisable to use an electric toothbrush, which offers advantages in the reduction of the plate index from 11 to 21% and in the reduction of gingivitis from 6 to 11% in the short and long term [56].

For optimal management, it is necessary to encourage pregnant women to implement a proper oral hygiene routine and to carry out 2–3 dental controls. In the first trimester, it is useful the control the pain and the prevention of the oral complications. During the months the patient continues to be treated more and more simply and without stress, it is useful to keep the patient in a sitting position and change the position often (to prevent lipothymia by compression of the vena cava). It is necessary to encourage the patient to use an electric toothbrush to reduce the plaque index. During pregnancy, in fact, the gums are more sensitive to local factors, such as plaque, calculus, prosthetics and overdue reconstructions. For this reason, there is a higher risk of gingivitis. 

The use of toothpastes with probiotics, even in pregnancy, can help restore a balanced oral microbiome, countering the onset of gingival diseases.

Therefore, if, in pregnancy, the levels of pathogenic bacteria increase, and in patients with COVID-19, these same bacteria are predominant in the samples of plaque, it would be useful to promptly establish the state of oral health of pregnant patients affected by COVID-19. Unfortunately, data on oral complications are not available to date in pregnant women suffering from this disease in the lower respiratory tract, and this is the main limitation of this review. Other limitations of the present report include the low number of patients in the selected studies, the presence of a limited number of bacteria and the lack of standardization of the oral hygiene procedures followed in the different reports.

Future research with more information and sufficient samples is desirable to understand better the clinical and microbiological changes in pregnant patients with COVID-19 compared with healthy pregnant patients. 

## 5. Conclusions

In light of the scientific literature, several oral changes have been noted in pregnant women, especially regarding the quality of subgingival bacterial plaque, which returns to favorable health conditions immediately after delivery. Increasing several bacterial species implicated in the development of gingival problems, such as *Fusobacterium nucleatum*, *Prevotella intermedia*, *Porphyromonas gingivalis* and *Aggregatibacter actinomycetemcomitans*, it is advisable to conduct professional oral hygiene sessions more frequently to lower the pathogenic bacterial load. Furthermore, these same bacterial species would be implicated in adverse pregnancy outcomes. 

Regarding the possible oral lesions associated with COVID-19 and any negative correlations associated with pregnant patients with SARS-CoV-2, the main data concerning changes in the level of the oral microbiome available to date have been reported. The focus of future research will be based on the evaluation of sample size and microbiological evaluation with long-term follow-up.

## Figures and Tables

**Table 1:** Articles related to the change of oral microbiome in pregnant women.

	Problem	Intervention/Comparison	Outcomes
Marwa Saadaoui et al., 2021	Changes in the oral microbiome during pregnancy	Analyze the bidirectional relationship between the oral microbiota and pregnancy	Oral dysbiosis, inflammatory cell activation and release of cytokines play a role in developing complications in pregnancy
Preethi Balan et al., 2018	Variations in oral microbial composition during pregnancy	Samples of saliva, subgingival plaque, plaque index and gingival index were validated in patients in the various trimesters of pregnancy and in patients in the post-partum period	The main species identified were *Porphyromonas gingivalis* (2.2%), *Treponema denticola* (1.10%), *Fretibacterium* spp. (0.67%) in subgingival and *Pevotella intermedia* plate samples (0.56%) in saliva samples
Anuradha Basavaraju et al., 2012	Variations in the anaerobic oral microbial flora in pregnant women before delivery and after delivery	Saliva samples were collected from pregnant women, before and after childbirth, and from pregnant women	The main anaerobic bacteria found in pregnant women were *Prevotella* spp., *Tannerella forsythia* and *Porphyromonas gingivalis*
Caroline Bearfield et al., 2002	Determine oral bacteria in the amniotic cavity	Dental plaque, vaginal swab and chorioamnion tissue in women attending for elective caesren section were evaluated	An association has been found between microbial DNA detection and cohmplication in pregnancy, including miscarriage, intrauterine death, neonatal death, preterm delivery and premature rupture of membranes
Priscila Viola Borgo et al., 2014	Assess a possible association between the periodontal condition and periodontal bacteria in pregnancy	Quantitative determinations of periodontal bacteria in women at the 2nd and 3rd trimesters of pregnancy and non-pregnant women were made	Pregnant women are more susceptible to gingivitis; the presence of *A. actinomycentemcomitans* in the biofilm sungingival could be considered for the treatment of periodontal disease.
Ana Carrillo-de-Albornoz et al., 2010	Determine whether gingival inflammation in pregnancy is related to a change in the subgingival biofilm	Microbiological, clinical and hormonal variables of pregnant women (evaluated every quarter and 3 months after delivery) and not pregnant women were evaluated	There were no microbiological differences in the trimesters of pregnancy, but only after childbirth; in addition, a correlation was established between the minor maternal hormone and *Porphyromonas gingivalis* and *Prevotella intermedia*
Mohammed Amir et al., 2020	Summarize findings related to the maternal microbiome.	Have been analyzed the most common infections in pregnant women, their effects on offspring and ongoing treatments	Perhaps there are common environmental factors in the oral cavity and in the placenta that favor colonization and growth of some bacteria, such as *Fusobacterium nucleatum*, which have negative effects on pregnancy
Wenzhen Lin et al., 2018	Evaluate changes in supragingival microbiota in pregnancy	Subgingival plaque and salivary hormones were evaluated in pregnant women and in the post-partum period, but also in patients not pregnant	*Neisseria* spp., *Porphyromonas* spp. and *Treponema* spp. are more present in pregnant women, while *Streptococcus* spp. and *Veillonella* spp. are more abundant in patients not pregnant
João Victor Silva Bett et al., 2019	Assess the prevalence of oral mucosal disorders during pregnancy.	Observational studies have been selected and the synthesis of the results has been calculated by the software	The overall prevalence of oral mucosal disorders was 11.8%. Gingival hyperplasia (17.1%), morsicatio buccarum (10%), oral candidiasis (4.4%), pyogenic granuloma (3%), and benign migratory glossitis (2.8%) were the most prevalent lesions.
Igor Jelihovschi et al., 2018	Evaluated the associations of subgingival counts of P. intermedia and P. gingivalis with the periodontal status of pregnant women	All the patients were subjected to periodontal clinical examination; BOP, PD, CAL and subgingival samples were collected	*Prevotella intermedia* is detected more frequently and in greater abundance in pregnant periodontitis patients.
Meital Nuriel-Ohayon et al., 2016	Describe microbial changes in pregnant women	Seven common bacteria species in the oral cavity of non-pregnant women and women in different pregnancy trimesters were compared	Microbial counts are higher in all stages of pregnancy, compared to the periosus of non-pregnancy; *Porphyromonas gingivalis* and *Aggregatibacter actinomycetemcomitans* are significantly higher in the early stages of pregnancy, compared to levels found in women not pregnant
Irene Yang et al., 2019	Explore possible associations among microbiome, periodontal inflammation and preterm birth	Saliva samples were analysed for interleukin-1 beta (IL-1β), metalloprotein-8 (MMP-8) and C-reactive protein (CRP) in patients with gingivitis and patients with healthy gums	No significant relationship was found between the subgingival microbiome, periodontal inflammation, and premature birth.

**Table 2:** The risk of bias of single studies. The green symbol means a low risk of bias and the yellow symbol means a moderate risk of bias.

	Adequate Sequence Generated	Allocation Concealment	Blinding	Incomplete Outcome Data	Registration Outcome Data
Marwa Saadaoui et al., 2021	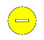	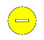	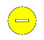	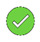	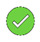
Preethi Balan et al., 2018	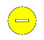	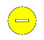	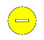	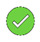	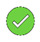
Anuradha Basavaraju et al., 2012	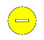	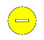	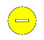	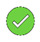	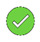
Caroline Bearfield et al., 2002	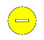	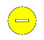	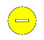	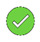	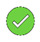
Priscila Viola Borgo et al., 2014	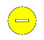	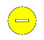	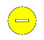	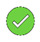	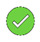
Ana Carrillo-de-Albornoz et al., 2010	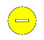	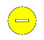	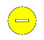	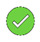	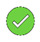
Mohammed Amir et al., 2020	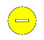	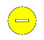	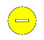	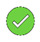	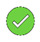
Wenzhen Lin et al., 2018	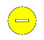	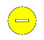	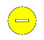	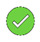	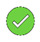
João Victor Silva Bett et al., 2019	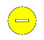	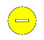	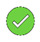	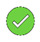	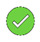
Igor Jelihovschi et al., 2018	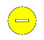	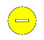	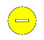	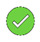	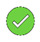
Meital Nuriel-Ohayon et al., 2016	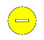	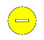	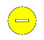	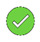	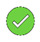
Irene Yang et al., 2019	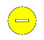	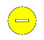	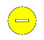	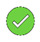	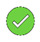

**Table 3:** The bacterial species that comprise the oral microbiome of a healthy patient, a pregnant patient and a COVID-19 patient.

Healthy Patient	COVID-19 Disease
*Steptococci*, *Lactobacilli*, *Staphylococci*, *Cotynebacteria* species make up the majority of the oral microbiome	High levels of *Fusobacterium nucleatum, Prevotella intermedia*
Pregnancy	
High levels of *Porphyromonas gingivalis, Treponema denticola, Prevotella intermedia, Tanerella forsythia, Aggregatibacter actinomycetemcomitans and Campylobacter rectus*	

## Data Availability

The data presented in this study are available on request from the corresponding author.
